# MAPK Pathways Coordinate Stress Adaptation by Mobilizing Specialized Gene Modules in Entomopathogenic Fungus *Beauveria bassiana*

**DOI:** 10.3390/jof11120839

**Published:** 2025-11-27

**Authors:** Shuaishuai Huang, Hailing Fan, Chenhua Zhu, Meixian Li, Leilei Liu, Mengdi Bai, Yonghong Zhou, Yongjun Zhang

**Affiliations:** 1 Key Laboratory of Biodiversity and Environment on the Qinghai-Tibet Plateau, Ministry of Education, School of Ecology and Environment, Tibet University, Lhasa 850000, China; s.huang1@utibet.edu.cn (S.H.); hailingfan4@126.com (H.F.); 18331537250@163.com (M.L.); lll1238@yeah.net (L.L.); bmd010605@163.com (M.B.); 2 Yani Wetland Ecosystem Positioning Observation and Research Station, Tibet University, Nyingchi 860000, China; 3 Key Laboratory of Agricultural Biosafety and Green Production of Upper Yangtze River, Ministry of Education, College of Plant Protection, Southwest University, Chongqing 400700, China; zhuu1998@163.com

**Keywords:** entomopathogenic fungi, *Beauveria bassiana*, gene co-expression network, transcriptomics, fungal stress biology, ergosterol

## Abstract

Mitogen-activated protein kinase (MAPK) cascades are critical for fungal development, stress adaptation. and virulence. However, their dynamic and stress-specific regulatory networks in entomopathogenic fungi remain largely unresolved. This study systematically investigates the roles of all three key MAPKs—BbHog1, BbSlt2, and BbMpk1—in insect pathogenic fungus *Beauveria bassiana*. A combination of detailed phenotypic profiling of deletion mutants (Δ*BbHog1*, Δ*BbSlt2*, and Δ*BbMpk1*) and time-course transcriptomics (RNA-seq at 0, 0.5, and 12 h) under osmotic, cell-wall, oxidative, and thermal stress conditions was employed, followed by weighted gene co-expression network analysis (WGCNA). This approach delineated twelve stress-responsive gene modules regulated by those MAPKs that were highly associated with fungal stress adaptation, including membrane repair, redox balance, cell-wall remodeling, and core metabolism. Functional analyses showed that Hog1 orchestrates osmoadaptation through coordinated control of osmolyte metabolism, glycolytic flux, and cell-wall remodeling; Slt2 protects against thermal damage by sustaining membrane integrity, ergosterol homeostasis, and redox balance; and Mpk1 directs oxidative stress responses by tuning mitochondrial activity, metabolic suppression, and detoxification pathways. In summary, this work outlines a concise, systems-level framework of MAPK-mediated stress regulation in *B. bassiana*, providing mechanistic insight into fungal environmental resilience and identifying molecular targets for the engineering of robust biocontrol strains.

## 1. Introduction

Mitogen-activated protein kinase (MAPK) pathways serve as multifaceted regulators of fungal biology, coordinating processes ranging from development and morphogenesis to stress adaptation, pathogenesis, and ecological fitness [1,2]. The three conserved MAPK pathways collaboratively orchestrate various aspects of fungal biology, while each also serves as a predominant regulator in response to distinct environmental cues and ecological niches. The Fus3/Kss1 branch governs developmental programs such as mating, filamentation, and appressorium formation, which are essential for host invasion [3], while the Hog1 and Slt2 cascades mediate osmotic, oxidative, and cell-wall stress conditions by activating glycerol accumulation, antioxidant defenses, and cell-wall remodeling [4]. In pathogenic fungi, the Pmk1/Fus3-like MAPK is indispensable for virulence, regulating cuticle penetration structures, toxin secretion, and sporulation during host colonization [5,6]. Crosstalk among MAPK branches further fine-tunes responses, ensuring coordinated deployment of protective circuits under multifactorial stress conditions [7,8]. The complex and interconnected regulation network mediated by MAPK cascades contributes greatly to fungal growth and survival in nature.

Entomopathogenic fungi are indispensable biological control agents in sustainable agriculture due to their specificity, environmental safety, and persistent insecticidal activity against diverse pest species [9,10]. Mitogen-activated protein kinase (MAPK) cascades are essential for enabling entomopathogenic fungi to infect host insects and endure adverse environmental conditions by maneuvering cellular responses against osmotic, oxidative, cell-wall, and thermal stress conditions [11,12,13]. In *Beauveria bassiana*, the Hog1 MAPK is critical for glycerol accumulation and antioxidant defenses under hyperosmotic and oxidative challenges, with Δ*Bbhog1* mutants exhibiting severe stress sensitivity and attenuated virulence in insect bioassays [14]. The Slt2 pathway governs cell-wall integrity and heat tolerance by regulating chitin synthases, membrane/cell-wall proteins, and membrane-repair genes, while its disruption leads to compromised conidiation and thermal sensitivity [13,15]. Moreover, Fus3/Kss1-like MAPKs orchestrate developmental switches—such as appressorium formation and invasive growth—which are essential for cuticle penetration in both plant and insect pathogens [5,12]. Although transcriptome and proteome studies have highlighted MAPK-responsive genes in entomopathogens and model fungi [12,16], a significant gap remains in the systematic, time-resolved transcriptomic analyses needed to map the dynamic, MAPK-mediated regulatory networks under multifactorial stress conditions.

Here, the sensitivity of three MAPK mutants under abiotic stress conditions was evaluated with detailed phenotypic profiling, and the regulatory role of those MAPKs in stress response was investigated with time-course RNA-seq (0, 0.5, 12 h) and weighted gene co-expression network analysis (WGCNA) to determine the stress sensitivity of each singular MAPK mutant. This approach uncovers a series of gene modules linked to core cellular processes and reveals both pathway-specific and shared regulons. The mobilization of suites of metabolic pathways by MAPK cascades responding to sensitive stress conditions was observed and verified with sets of fluorescent observations, enzymatic activity tests and qRT-PCR. By elucidating the temporal and network-level architecture of Hog1, Slt2, and Mpk1 signaling under sensitive stress conditions, our study provides a dynamic blueprint for fungal adaptation and virulence. This knowledge not only advances basic understanding of MAPK function in entomopathogenic fungi but also informs the rational design of hyper-resilient biocontrol strains.

## 2. Materials and Methods

### 2.1. Fungal Strains and Culture Condition

Wild-type *B. bassiana* strain Bb0062 (denoted as WT) isolated from cadavers of *Plutella xylostella* larvae was identified and deposited in the China General Microbiological Culture Collection Center (CGMCC, accession number CGMCC 7.34), and MAP-kinase derivative mutant strains Δ*BbHog1*, Δ*BbMpk1*, and Δ*BbSlt2* were constructed previously [5,14,15]. Those fungal strains were grown on Czapek-dox agar/broth (CZA/B) (Difco Laboratories, Detroit, MI, USA), potato dextrose agar/broth (PDA/PDB) (Difco Laboratories, Detroit, MI, USA), Sabouraud dextrose agar or broth (SDA/SDB) (Difco Laboratories, Detroit, MI, USA), or SDA/SDB (Difco Laboratories, Detroit, MI, USA), supplemented with 1% (*wt*/*vol*) yeast extract (SDAY/SDBY) as indicated. Unless otherwise indicated, for use as inoculation, conidia were collected from SDAY plates after incubation for 14 days at 26 °C (15 h light: 9 h dark cycle) and subjected to downstream experiments.

### 2.2. Phenotypic Assays

Colony growth and stress response tests were performed as previously detailed [17]. Briefly, conidia were collected from SDA plates after 10 d of cultivation, the concentration was adjusted to 10^7^ conidia/mL after counting using a hemocytometer, and 100 μL aliquots were uniformly spread on SDAY plates covered with cellophane sheets (90 mm in diameter). Hyphal plugs (diameter = 5 mm) were taken from the SDAY plates after 3 d incubation (26 °C) and placed in the middle of fresh test plates. Test plates included SDA and CZA amended with 1.0 M sorbitol, 16 μM of menadione (MND), or 25 μg/mL Congo red (CR). In indicated cases, a supplement of ergosterol (C_28_H_44_O, 2.5 mM) was added during MAP-kinase stress response tests to assess their ability to rescue observed phenotypes, particularly in the ∆*BbSlt2* mutant. Unless otherwise indicated, plates were incubated at 26 °C with a 15:9 h light: dark cycle. Colony diameters were measured daily. Relative growth inhibition (RGI) was calculated using the following formula: RGI = (Dc − Ds)/(Ds − d) × 100, where Dc and Ds are the mean diameters of at least 3 fungal colonies in the control and stress treatments, respectively, and d denotes the diameter consistence of the primary plug (5 mm) [18]. All the RGI data was visualized with a radar plot generated by the ggplot2 R package. All the experiments were performed in triplicate and repeated three times with independent batches of conidia. Aerial hyphae of WT and three MAPK mutants grown on SDAY plates for 3 days were collected and subjected for scanning electron microscopic (SEM) (Hitachi, Tokyo, Japan) observation.

### 2.3. Transcriptomic Analyses and qRT-PCR Validation

Samples for RNA-seq analyses were prepared as previously described [18]. Briefly, wild-type *B. bassiana* and MAP-kinase mutants were inoculated into SDB broth at an initial conidial concentration of 10^5^ conidia/ml. Fungal (hyphal) cultures were collected after 3 days of growth and washed in SDB media containing the indicated stress agent, including (i) 1.0 M sorbitol, (ii) 16 μM of MND, 25 μg/mL of CR, and (iv) SDB incubated at 32 °C. Two time points of induction in the presence of the stress agent—namely, 30 min and 12 h—were adopted for each test condition. Fungal cells were collected by centrifugation and washed with 20 mM PBS (phosphate-buffered saline, pH 7.3), and total RNA was isolated using TRIzol reagent (Invitrogen Life Technologies, Carlsbad, CA, USA), following the manufacturer’s instructions. Cultures of wild-type and MAPK mutants of *B. bassiana* (strain 0062) were grown for each tested condition with three replicates and mixed evenly for RNA extraction. RNA purity and quality were examined via agarose gel electrophoresis and photometric analysis (Pearl nanophotometer, Implen, Munich, Germany). RNA-Seq libraries were generated using a Truseq^TM^ RNA sample prep Kit (Illumina, San Diego, CA, USA) following the manufacturer’s instructions. Constructed cDNA libraries were subjected to RNA sequencing using an Illumina HiSeq™ 2000 (Majorbio, Shanghai, China) with paired-end sequencing mode for the generation of transcriptomic data bearing an average sequence length of 150 bp. A widely used transcriptomic analysis pipeline [19] was implemented for the processing of gene expression data through the following steps: RNA-Seq data were initially subjected to FastQC (v 0.11) for sequencing quality control, followed by Trimmomatic (v 0.32) for sequence adaptor trimming and removal of unpaired sequences. Then, Hisat2 (2.2.1) was used for mapping of transcripts to the genome of *Beauveria bassiana* strain ARSF 2860. Samtools (v 0.1.18) and Subread (v 2.0.0) were used for transcript assembly and transcript abundance quantification. All analyses were performed on a Tecent cloud server with a Linux system running Ubuntu 18.04.4. The raw RNA-seq data were deposited in the NCBI Gene Expression Ominibus with series accession number PRJNA865046.

A small amount of mycelium (WT/Δ*BbHog1*/Δ*BbMpk1*) was inoculated into 100 mL of SDAY medium and incubated on a shaker for 4 days. The mycelium was then filtered out, transferred to the corresponding induction medium, and re-filtered to obtain the experimental mycelium. RNA was extracted from the induced mycelium using Biosharp Total RNA Isolation Reagent and an Aidlab FASTeasy Universal Plant & Fungi RNA Kit (according to the manufacturers’ manuals). cDNA was synthesized via reverse transcription (following the Thermo Scientific REvertAid First Strand cDNA Synthesis Kit protocol, Waltham, MA, USA). qPCR was performed using Servicebio^TM^ 2 × SYBR Green qPCR Master Mix (Wuhan, China) according to the provided instructions. The amplification program consisted of 95 °C for 2 min, followed by 39 cycles of 95 °C for 5 s, 60 °C for 30 s, and a melting curve from 65 °C to 95 °C with a temperature increase of 0.5 °C per 5 s. Actin (GenBank ID: HQ232398) was used as the internal reference gene. A list of the primers used in the study is presented in Supporting Information Table S1.

### 2.4. Analyses of the Differentially Expressed Genes (DEGs) in MAPK Mutants

Transcript counts were first filtered by removing genes with 0 counts in the data for the wild-type and mutant strains, then transformed into FPKM (Fragments Per Kilobase of transcript per Million mapped reads) values with R package DESeq2 (v 1.36.0). A value of 1 was added to all FPKM values (FPKM + 1); then, the values were transformed with a log2-based algorithm and quantile normalization to correct data bias and generated DEGs in stress (st) as compared to the normal condition with the following formula: Log2((FPKM^st^ + 1)/(FPKM^nor^ + 1)). A filtration of the expression ratio was performed such that genes with |Log2FC| ≥ 2 were screened as DEGs and those with|Log2FC| ≤ 0.5 were categorized as silent genes. The differential expression genes in MAP-kinase mutants responding to abiotic stress were evaluated according to a curated algorithm to identify upregulated DEGs in MAPK mutants responding to the given stress, i.e., DEGs^Up^ = Log_2_FC(WT^st^/WT^nor^) ≤ −2& Log_2_FC(ΔMAPK^st^/ΔMAPK^nor^) ≥ −0.5), and downregulated DEGs, i.e., DEGs^Dn^ = Log_2_FC(WT^st^/WT^nor^) ≥ 2& Log_2_FC (ΔMAPK^st^/ΔMAPK^nor^) ≤ 0.5.

### 2.5. Global Gene Co-Expression Analysis

A pair of R-based tools—Coseq (1.20.0) [20] and WGCNA [21]—and a hierarchical clustering method (Pearson) were employed for gene co-expression analysis. Briefly, extracted transcript counts from all constructed transcriptomes were subjected to Coseq in R for normalization, with Log-Centered Log Ratio (LogCLR) transformation as an expression index indicating transcript abundance in RNA-seq samples. A co-expression compendium was constructed with the k-means clustering algorithm after TMM normalization of LogCLR-transformed reads. Co-expressed genes across the 18 sets of transcriptomic data were clustered and formed 80 modules. For generation of the co-expression networks, the WGCNA protocol was employed. In brief, FPKM values of all 18 transcriptomes were subjected to the online WGCNA package in R. After sample-type classification, hierarchical clustering was conducted to classify genes with similar expression profiles that generated 12 modules after optimization of the correlation threshold of gene–gene co-expression. All of the 12 modules with co-expression degree and gene information were exported and subjected to Cytoscape (v 35.0) and Gephi (v 0.10.1) for generation of co-expression networks with deliberate decorative editing of modular gene functions.

### 2.6. Fluorescent Observation and Glycolysis Stress Assay

For the mitochondrial membrane potential test, a small amount of mycelium (WT/Δ*BbHog1*/Δ*BbMpk1*/Δ*BbSlt2*) was inoculated into 100 mL of SDY medium and incubated on a shaker for 4 days. The mycelium was filtered through a wire mesh, and the conidial suspension was collected. The suspension was concentrated by centrifugation at 4 °C and 4000 rpm using a refrigerated centrifuge, and the supernatant was discarded. The WT and ΔMpk1 mycelium were washed with 50 mL of 16 μM menadione in SDY medium and induced for 30 min. After washing the culture three times, the mycelium was resuspended to prepare a spore suspension. Staining was performed using a Mitochondrial Membrane Potential Assay Kit with JC-1 (Solarbio, Beijing, China) and following the corresponding instructions. Stained cells were visualized under an excitation wavelength of 515 nm and emission wavelength of 529 nm for mitochondrial monomers, and aggregates were observed with an excitation wavelength of 585 nm and emission wavelength of 590 nm with a confocal microscope (Leica, Wetzlar, Germany).

Fungal cell-wall staining was performed with Fluorescein 5-isothiocyanate (FITC)-labeled wheat germ agglutinin (WGA) and Concanavalin A (ConA) (Maokangbio, Shanghai, China). Cell suspensions were prepared same as described above. The collected blastospore suspension was washed by centrifugation and fixed with 4% paraformaldehyde at room temperature for 1 h, followed by two washes with PBS. A 50 µL aliquot of the resuspended sample was mixed with 100 µL of 20 µg/mL FITC-ConA/FITC-WGA and incubated at room temperature for 1 h, then washed and resuspended in PBS. FITC-ConA/FITC-WGA-stained cells were observed with a confocal microscope (Leica, Wetzlar, Germany) (excitation:494 nm; emission: 520 nm).

For glycolysis stress fluorometric assays, cell suspensions were prepared as described above. The collected blastospore suspension was washed by centrifugation and resuspended in PBS. The cell concentration was calculated and balanced to 10^6^ cells/mL. The assays were performed following the protocol of the Glycolysis Stress Fluorometric Assay Kit (Solarbio, Beijing, China). Briefly, measurement wells, 2-DG control wells, and blank wells were set up ahead of time. A volume of 100 µL of the cell suspension was added to each corresponding well of a black, clear-bottom 96-well microplate. The plate was incubated in a non-CO_2_ environment at 25 °C in the dark for 30 min. The fluorescence microplate reader was set to 25 °C. Then, 100 µL of working assay solution was added to the measurement and blank wells. For the 2-DG control wells, 10 µL of reagent 5 and 90 µL of glucose-free working solution were added. Fluorescence was measured at an excitation wavelength of 490 nm and emission wavelength of 535 nm with a Fluorometric Microplate-Reader (Tacan, Männedorf, Switzerland), and readings were taken every 4 min over 100 min. The extracellular acidification rate (ECAR) was calculated accordingly.

### 2.7. Data Analysis and Visualization

For transcriptomic data similarity analysis, transcript reads from all 18 samples were collected and subjected to R package “ape” (v 5.6) for generation of a PCoA plot, and the hierarchical clustering visualization tool in the Omicshare platform was used for RNA-seq sample data clustering. DEG overlap datasets comparing stress conditions were analyzed by collecting DEG groups under 6 stress conditions and subjected to the nVenn tool for generation of a Venn plot [22]. Gene ontology and KEGG analyses were used for functional classification of given subsets of DEGs or co-expressed gene modules, with genes subjected to GO/KEGG classification tools in the Omicshare platform (www.omicshare.com) with Uniprot-annotated GO terms and KEGG BlastKOALA-mapped terms of the *B. bassiana* genome as references. Functional enriched pathways were visualized with a dot plot or heat map (R package ggplot2). Enriched pathways were also regenerated and visualized manually based on KEGG pathway collections. For other gene expression visualization plots, a either violin plot (R package ggplot2) or bar plot in Sangerbox 3.0 was employed. For all experimental data, at least three replicates were analyzed, and the mean value, standard deviation. (*t*-test) were collected for data visualization.

## 3. Results

### 3.1. Distinct Phenotypes of BbHog1, BbSlt2, and BbMpk1 Mutants in the Presence of Series Stressors

**Figure 1:**
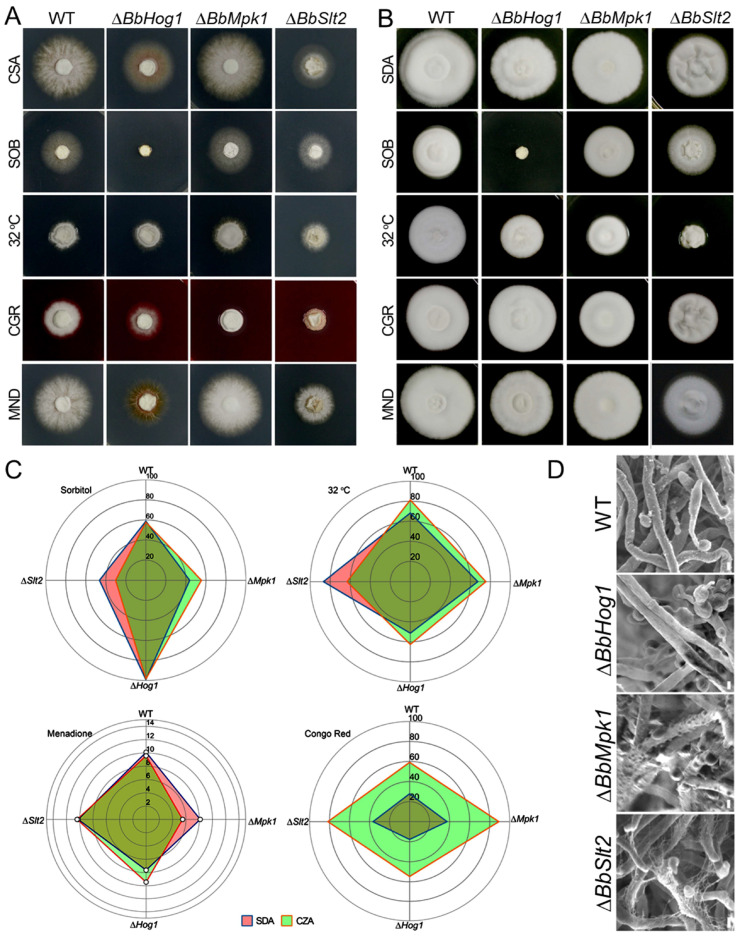
Stress sensitivity profiles and morphological phenotypes of MAPK mutants. Colony growth of the WT strain and 3 MAPK mutants (∆*BbHog1,* ∆*BbMpk1*, and ∆*BbSlt2*) under osmotic stress (1.2 M Sorbitol); stress induced by cell wall-perturbing agents, i.e., Congo red (CGR, 50 µg/mL); oxidative stress (menadione, MND 16 µM); and heat stress (32 °C) on SDA and Czapek-Dox agar plates. Colony growth of MAPK mutants on CZA (**A**) and SDA (**B**) was photographed after 10 days of growth. (**C**) Radar plot of relative growth inhibition (RGI) of MAPK mutants. (**D**) scanning electron microscopic observation of fungal aerial hyphae of the WT strain and three MAPK mutants (bar indicates 5 μm).

To investigate the versatile role of MAP kinases in fungal adaptation to diverse stress conditions, the colony growth of wild-type *B. bassiana* and three MAP-kinase mutants was assayed in media supplemented with a series of abiotic stress agents as detailed in Section 2 (Figure 1A,B). The relative growth inhibition (RGI) of MAPK mutants under abiotic stress was visualized as a radar plot (Figure 1C). Mutation of three MAPK genes showed distinct response to different kinds of stress. In fronting of osmotic stress, Δ*BbHog1* exhibited severely impaired growth and showed the most pronounced sensitivity to osmotic stress with severe RGI of 99.04% on SDA and RGI of 98.46% on CZA plates as compared to the WT strain (RGI of 58.69% on SDA and 57.75% on CZA), while minor growth inhibition was seen for two other MAPK gene mutants (RGI of 32.26~55.45%). With the given heat stress, the growth inhibition of all three MAPK gene mutants was minorly relieved (RGI: 62.57~75.43%) as compared to the WT strain, which yielded RGI of 81.23% on CZA plates at 32 °C. Rich nutrition in SDA medium greatly attenuated heat stress for the WT (RGI: 68.25%), Δ*BbHog1* (RGI: 51.37%), and Δ*BbMpk1* (RGI: 67.38%) strains, but the Δ*BbSlt2* mutant was slightly rescued with significant growth inhibition on SDA (RGI: 87 ± 2.5%)—nearly 20% higher than that of the WT strain. In tests of cell-wall perturbing stress (Congo red), all four strains showed a coordinated response on CZA and SDA plates. RGI was greatly elevated for the Δ*BbMpk1* (CZA/SDA: 9.64%/30.00%) and Δ*BbSlt2* (CZA/SDA: 8.73%/22.40%) strains as compared to the WT strain. After exposure to oxidative stress, Δ*BbMpk1* showed an opposite response, with elevated tolerance on both SDA (1.95%) and CZA (4.08%) plates as compared to the WT parent. This observation is consistent with previous findings [23]. Similar RGI values were seen for Δ*BbSlt2* grown on SDA/CZA plates compared to the WT strain. Δ*BbHog1* showed similar RGI values to WT strains on CZA and SDA plates under cell-wall perturbing stress (Congo red) and oxidative stress (MND). These data revealed distinct growth variations of MAP-kinase mutants responding to a series of abiotic stresses.

The fungal cell wall was found to play key role in fungal abiotic stress resistance. SEM observations revealed that knockout of individual MAP kinases led to dramatic alterations in cell surface morphology (Figure 1D). Deletion of Hog1 resulted in relatively smooth hyphal surfaces as compared to the dense, fine-scaled protrusions on the surfaces of WT hyphae. Hyphae of Δ*BbMpk1* developed abnormally serried small bumps across the cell surface. Furthermore, ablation of BbSlt2 gave rise to an unusual filiform texture that covered the mycelia. To visualize the changes in the fungal cell wall under abiotic stress conditions, we employed FITC-labeled lectins—concanavalin A (ConA), which binds specifically to α-mannopyranosyl and α-glucopyranosyl residues, and wheat germ agglutinin (WGA), which targets N-acetylglucosamine-containing carbohydrates)—to monitor alterations in cell-wall composition between the wild-type (WT) strain and three MAPK mutants (Figure S1A). Under the same light intensity, the WT strain exhibited the strongest ConA fluorescence, whereas the three MAPK mutants showed relatively weaker ConA signals, suggesting reductions in α-mannopyranosyl and α-glucopyranosyl glycoproteins compared to the WT parent. WGA lectin labeling showed that the Δ*Bbslt2* and Δ*Bbhog1* mutants exhibited weaker WGA fluorescence than the WT strain under the same light intensity. In contrast, the Δ*Bbmpk1* mutant produced a denser and brighter WGA signal.

### 3.2. Global Transcriptomic Responses of B. bassiana and MAP-Kinase Mutants to Challenge by Osmotic, Oxidative, Cell Wall-Perturbing, and Heat Stress

To pinpoint the underlying mechanism by which MAP kinases control abiotic stress responses, the global gene expression profile was evaluated in a time-series course (0 h, 0.5 h, and 12 h) with a focus on the responses of the MAP kinases to stress conditions, e.g., hypersensitivity of ∆*BbHog1* to osmotic stress, increased sensitivity of ∆*BbSlt2* to heat stress, and increased tolerance of ∆*BbMpk1* to oxidative stress. Fungal strains treated with abiotic stress were subjected to RNA extraction and RNA sequencing as previously described in Section 2. All treated samples were prepared in triplicate and mixed for sequencing. Sequenced RNA reads were successfully annotated, with an average of 80% of the raw reads mapped to the *B. bassiana* ARSF2860 genome, corresponding to 20 million reads. After filtration of low-read (counts (0) > 12) genes, 9149 mapped genes were subjected to further differential gene expression/co-expression analyses.

**Figure 2:**
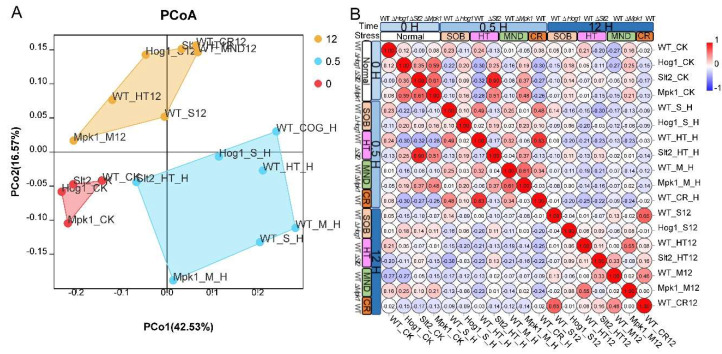
Principal coordinate analysis (**A**) and hierarchical clustering (**B**) of collected RNA-Seq samples. Sample names were assigned as Strain_Stress_Time. Strains WT, ∆*Mpk1*, ∆*Hog1*, and ∆*Slt2*; stress agents: Heat (HT), Sorbitol (S), Menadione (M), and Congo red; time series: zero (Blank), 30 min (H), and 12 h (12).

In Pearson correlation analysis of RNA-seq samples, we found samples derived from different test conditions were clustered in a certain pattern, while those collected at the same time point (i.e., 0, 0.5, or 12 h) were tightly packed (Figure 2A). These analyses indicated a closer correlation in terms of stress-treated time rather than variations in stress agents or MAP-kinase mutation strains. A hierarchical correlation matrix analysis also revealed certain correlations between RNA-seq samples (Figure 2B). Hierarchical analysis showed that ablation of each MAP kinase considerably changed the global gene expression profiles of *B. bassiana* in SDB with or without acute or sustained osmotic/oxidative/heat stress treatment, with a low R^2^(<0.12) correlation of the WT strain with MAPK mutants under specialized stress conditions, except for the WT and ∆*BbMpk1* strains in acute oxidative stress treatment (R^2^ = 0.61) (Figure 2B). Two time sets of stress treatment (0.5 h and 12 h) greatly changed gene expression profiles for all test strains, with a weak correlation (R^2^ ≤ 0.51) under normal conditions, except ∆*BbSlt2* under heat stress, which showed tight a correlation with untreated ∆*BbSlt2* (R = 0.90), indicating the deletion of BbSlt2 desensitized fungal response to heat stress. Interestingly, among those tight-correlation samples (R^2^ ≥ 0.6), few strains showed similar RNA-seq profiles under varied time–stress conditions: (i) WT presented similar transcriptomes under heat and cell wall-perturbing stress with 30 min treatment (R^2^ = 0.83), but elongated stress treatment (12 h) erased this similarity, with R^2^ = −0.03. (ii) The WT strain responded similarly under 12 h treatment osmotic and cell wall-perturbing stress (R^2^ = 0.65). The WT and ∆*BbMpk1* strains showed a medium correlation (R^2^ = 0.61) in the short term (0.5 h) under oxidative stress treatment.

### 3.3. Co-Expression Analysis Revealed the MAP Kinases Mediate Key Gene Modules Responding to Abiotic Stress

**Figure 3:**
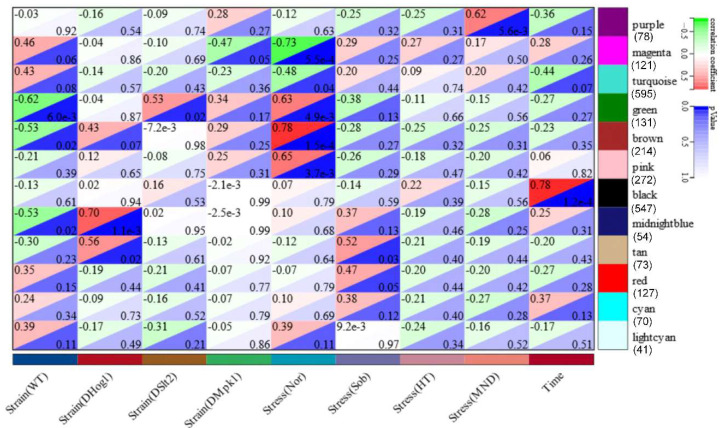
Module–trait association analysis with hierarchical clustering algorithm. WGCNA generated 12 co-expression modules for RNA-Seq sample variables (strain, stress, and time). Upper-left triangles indicate co-expression efficiency, while lower-right triangles refer to *p*-values.

To globally uncover the MAPK-mediated networks in response to abiotic stress, we performed Weighted Gene Co-expression Network Analysis (WGCNA). This identified gene co-expression networks (GCNs) associated with stress adaptation and key mobilized pathways. In total, 4646 genes that were differentially expressed in at least one of the MAPK mutants were subjected to co-expression analyses. Hierarchical clustering analysis of the relationship between enriched modules and sample traits (strain types/stress/time variations) was conducted (Figure 3). The expression patterns of bundle gene sets were tightly correlated strain types/stress/time variations. Collectively, 8 out of 12 modules showed tight correlation with the sample traits (R^2^ ≥ 0.5) and harbored 1490 genes with distinct expression profiles. Of those, three modules yielded both significant positive and negative correlations with different sample traits, while four modules showed high positive correlations with the sample traits. The non-stress trait exhibits highest positive and negative correlated modules (brown, R^2^ = 0.78, *p* = 1.5 × 10^−4^; magenta, R^2^ = −0.73, *p* = 5.5 × 10^−4^).

**Figure 4:**
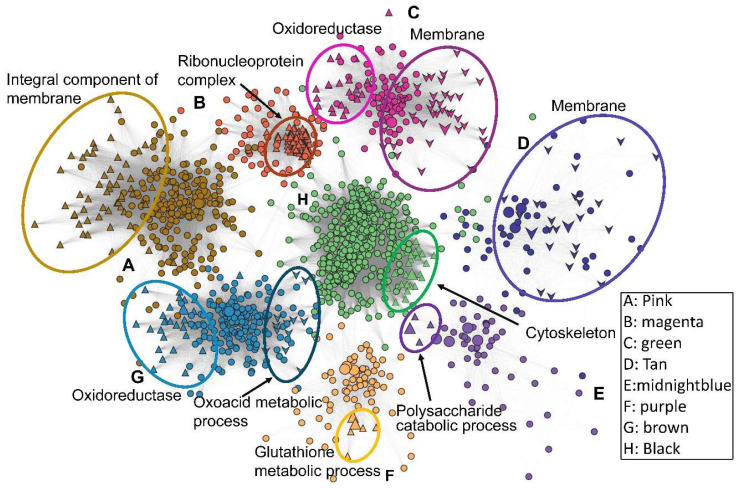
Co-expression network of DEG modules generated by WGCNA. Genes in different modules (in Figure 3) are colored accordingly: A, pink; B, magenta; C, green; D, tan; E, midnight blue; F, purple; G, brown; H, black. Functional GO-enriched subgroups are circled and labeled with different shapes. Node size indicates co-expression intensity relative to neighboring genes.

To further annotate the module gene function and the module–trait correlations, a co-expression network of the eight modules were generated, along with stacked Gene ontology (GO) marks of module genes (Figure 4). Global expression analysis showed that the green module was negatively regulated in the WT strain with/without stress treatments, and 28.24% of genes in the green module are membrane proteins, while 16% of genes encode oxidoreductases. The brown module harvested 29 oxidoreductase genes and 13 genes in oxoacid metabolism. Of the 201 genes in the pink module with high correlation with the non-stress trait, 57 genes encode an integral component of the membrane, 7 genes are involved in hexose metabolism, and 7 members are extracellular proteins. The magenta module, which was relatively active/depressed under non-stress conditions, enriched 121 genes, 68 of which encode the ribonucleoprotein complex. Also, ∆*BbHog1* harbors one positive related module (R2 = 0.7, *p* = 1.1 × 10^−3^) (midnight blue), with 54 genes upregulated uniformly in ∆*BbHog1* with/without stress treatments as compared to all other samples, indicating a Hog1-dependent gene expression suppression profile, including three Cyp450s (BBA_07811, BBA_07828, BBA_00881), three chitinase/chitosanase genes (BBA_00297, BBA_02230, BBA_06270), two members (BBA_07299, BBA_07814) associated with glycerophospholipid metabolism, and 18 genes annotated as virulence factors with the PHI prediction. The black gene module is highly time course-dependent, covering 547 differentially expressed genes in all strains after stress treatments (30 min or 12 h). These genes were largely enriched in fundamental metabolic processes, e.g., cytoskeleton remolding (27 genes) (GO, *p* <0.001), cell-cycle processes (27 genes) (KEGG, *p* < 0.001), glycolysis (10 genes) (KEGG, *p* < 0.01), and the MAPK signal pathway (11 out of 66 genes) (KEGG, *p* < 0.01). The presence of oxidative stress positively activated the purple module (R^2^ = 0.6, *p* = 5.6 × 10^−3^), enriching 78 genes in which the ROS detoxification process is largely activated, e.g., 6 genes enriched in glutathione metabolism (*p* < 0.001), 20 genes in the oxidoreductase response, 5 genes in response to oxidative stress (*p* < 0.001), 8 enriched genes as members of sulfur compound metabolism (*p* < 0.001), and 20 enriched genes encoding membrane proteins (*p* < 0.001).

### 3.4. Master Regulatory Role of BbHog1 in Fungal Respondse to Osmotic Stress

**Figure 5:**
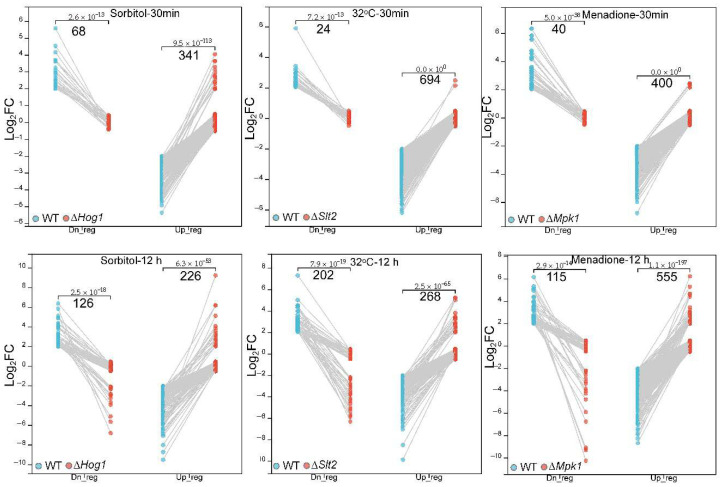
Relative expressions of DEGs in all three MAPK mutants under sensitive stress conditions. Dots in plots indicate DEGs in a given group. Dn_reg, downregulation; Up_reg, upregulation. The WT strain is indicated in blue, and MAPK mutants are colored red.

**Figure 6:**
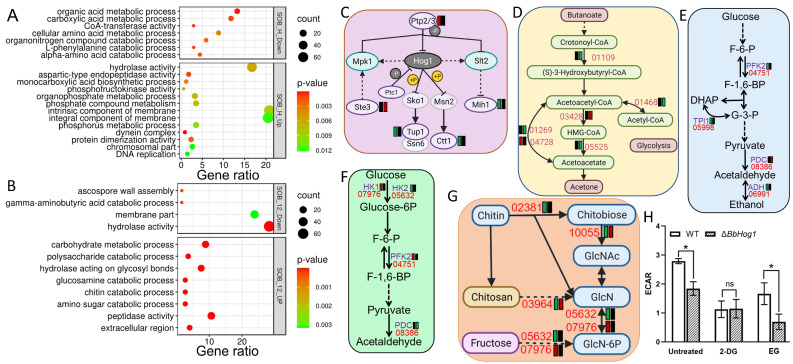
Gene ontology functional classification and enriched KEGG pathways of DEGs in WT and ∆*BbHog1* strains under osmotic stress treatment. GO classification of DEGs in ∆*BbHog1* as compared to the WT parent under 30 min (**A**) or 12 h (**B**) osmotic stress when normalized to non-stress conditions. Up/Down in gray box indicates relative upregulation/downregulation in ∆*BbHog1* relative to WT under sorbitol stress, both normalized to non-stress treatment, and C-E indicates the relative expression of DEGs in the MAPK signaling pathway (gene tagged as yeast homolog). (**C**) Ketone body metabolism (**D**) and glycolysis pathway (**E**) under acute osmotic stress. (**F**,**G**) refers DEGs in the glycolysis pathway and the chitosan biosynthesis pathway under 12 h sorbitol stress, respectively. In (**C**–**G**), two rectangles beside the gene ID of DEGs refer to upregulation (red), no change (black), or downregulation (green) in WT and ∆*BbHog1* as compared to strains under non-stress conditions. Gene IDs are indicated in red with the omission of the “BBA_” prefix. Pathways were reconstructed according to the KEGG pathway database and YeastPathways. (**H**) fluorometric glycolysis stress assessment of WT and ∆*BbHog1* strains treated with transient osmotic stress. ECAR refers to the extracellular acidification rate, 2-DG refers to 2-Deoxy glucose as a competitive hexokinase inhibitor, and ER refers to the efficiency of glycolysis. * *p* < 0.05; ns for non-significance.

The differential expression genes in MAP-kinase mutants responding to abiotic stress were analyzed as described in Section 2 to identify up/downregulated DEGs in MAPK mutants responding to acute or sustained stress. All such DEGs were visualized using paired dot plots, in which down- and upregulated genes were annotated separately and six paired groups (SOB-0.5, SOB-12, MND-0.5, MND-12, HT-0.5, and HT-12) were generated to show DEGs of MAP-kinase mutants versus WT strains under abiotic stress in a given time course (Figure 5, Tables S2–S4). MAP-kinase mutant ∆*BbHog1* harbored 409 DEGs (Up:68/Dn:341) and 352 DEGs (Up:126/Dn:226) under acute (0.5 h) and sustained (12 h) osmotic stress treatments, respectively (Figure 5, Table S2). Functional enrichment analysis (GO/KEGG) showed that depletion of *BbHog1* greatly disrupts fungal mobilization of fundamental metabolic pathways, including cell membrane integrity, polysaccharide metabolism, carbohydrate metabolism, and amino acid (tryptophan, valine/leucine/isoleucine) metabolism in response to acute or sustained osmotic stress (Figure 6). GO enrichment analysis showed that, with acute (0.5 h) osmotic stress treatment, the organic acid (mainly amino acid) metabolism was significantly attenuated, while membrane-associated proteins and hydrolases (49, mainly glycosidases) were relatively upregulated in ∆*BbHog1* (Figure 6A). Metabolic pathway enrichment (KEGG) analysis indicated that three pathways were significantly affected by ablation of ∆*BbHog1*: (i) The lipid metabolism engine and alternative energy source, i.e., the ketone body metabolism, was relatively downregulated in ∆*BbHog1* under acute osmotic stress (Figure 6D), including coenzyme A transferase, BBA_04728, and other key metabolic genes (BBA_01109, BBA_01468, BBA_01269, and BBA_05525). (ii) The MAPK signaling pathway fluctuated with the loss of expression suppression of two downstream Hog1 regulators (Tup1/Ctt1), while the expression the other three components in the MAPK pathway was abnormally elevated or silent, in contrast to the WT strain, in response to acute osmotic stress (Figure 6C). The expression of the glycolysis pathway was also disturbed with loss of BbHog1, with key enzymes downregulated in the WT strain while silent in ∆*BbHog1* under acute osmotic stress (Figure 6E). Interestingly, the glycolysis stress assay showed that ∆*BbHog1* yields an attenuated glycolytic capacity as compared to the WT strain under acute osmotic stress (Figure 6H). The extracellular acidification rate (ECAR) of ∆*BbHog1* decreased by approximately 34% compared with the wild-type (WT) strain. Upon the addition of 2-deoxy-D-glucose (2-DG), a competitive inhibitor of hexokinases, the ECAR of the WT strain, was greatly decreased compared to ∆*BbHog1*. Collectively, the WT strain exhibited a 58% higher ECAR (mediated by hexokinases) than the ΔBbHog1 mutant, indicating that BbHog1 is crucial for maintaining efficient glycolytic flux and respiratory activity. In addition, three dynein microtubule motor proteins (BBA_00304, BBA_05442, and BBA_05542) involved in cell skeleton dynamics were abnormally upregulated in ∆*BbHog1* under acute osmotic stress. Two upregulated genes (BBA_01294 and BBA_00889) and two downregulated genes (BBA_04972 and BBA_08551) in ∆*BbHog1* as compared to the WT parent in response to acute osmotic stress were randomly selected for qRT-PCR validation. The expression profiles of the four tested genes obtained via qRT-PCR were consistent with the results of our transcriptomic analysis (Figure S1C).

In the 126 downregulated DEGs in the ∆*BbHog1* mutant responding to 12 h osmotic stress treatment (SOB_12_Dn) (Figure 5, Table S2), the expression of 31 membranal protein-encoded genes was greatly attenuated, encoding 26 membrane transporters (annotated with TCDB), including 7 ion transporters. Two out of three genes in the spore-wall assembly process were also depressed in ∆*BbHog1*(Figure 6B). Furthermore, catabolism of abiotic stress alleviators was attenuated, two out of three key genes downregulated in the MAP-kinase mutant. The key carbohydrate catabolic pathway—glycolysis—was largely depressed, with three key genes relatively downregulated in ∆*BbHog1* as compared to the WT parent (Figure 6F). Of the 226 DEGs upregulated in the ∆*BbHog1* mutant under sustained osmotic stress treatment as compared to the WT strain, fungal cell-wall integrity and catabolism were greatly influenced, including chitin/polysaccharide catabolism (8 genes), membrane transporters (17), hydrolases (64), and peptidases (24) (GO, *p* < 0.01) (Figure 6B). Five key genes enriched in chitosan biosynthesis to rescue cells were remedially upregulated in ∆*BbHog1* under consistent osmotic stress (Figure 6G). Interestingly, three genes enriched in the glycerophospholipid metabolism pathway were collectively upregulated in ∆*BbHog1* in response to acute and sustained osmotic stress treatment, including two phospholipase C (BBA_03202, BBA_07286) and one phosphatidylserine decarboxylase (BBA_10177). The expressions of 1 succinate dehydrogenase (BBA_09596) gene in the TCA cycle and 10 peptidase genes were also elevated in ∆*BbHog1* in response to osmotic stress (30 min/12 h) as compared to the WT parent.

### 3.5. Deletion of BbSlt- Paralyzed Suites of Pathways Responsible for Fungal Tolerance to Heat Stress

**Figure 7:**
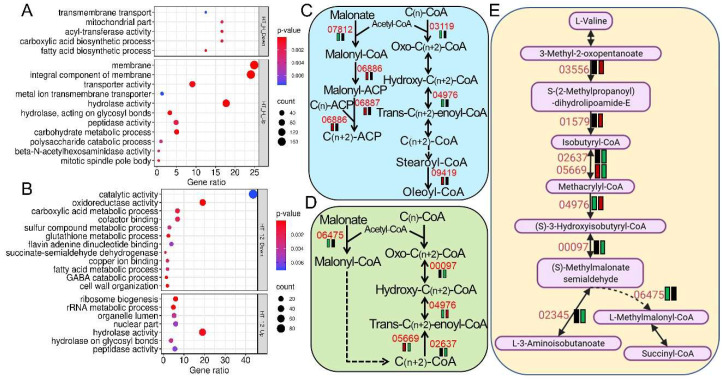
Go functional enrichment and illustration of over-represented KEGG pathways of DEGs in the WT strain and ∆*BbSlt2* amended with heat stress. Go annotation of DEGs in ∆*BbSlt2* as compared to the WT parent under 12 h (**A**) or 30 min (**B**) of osmotic stress when normalized to non-stress conditions. (**C**,**D**) Relative expression of DEGs in the fatty acid metabolism pathway under 30 min and 12 h sorbitol stress treatment, respectively. (**E**) Relative expression of DEGs in Val/leu/Ile degradation under 12 h sorbitol stress when compared to non-stress conditions. Annotations of expression and gene IDs are the same as in Figure 6.

The ablation of BbSlt2 greatly attenuated fungal tolerance to heat stress (Figure 1). The comparative transcriptomic analysis also revealed that suites of DEGs and pathways were responsible for ∆*BbSlt2* sensitivity to heat stress. Collectively, 718 DEGs were generated in the ∆*BbSlt2* mutant, in which 96.7% of DEGs (694) were upregulated as compared to the WT parent bearing acute heat stress (Figure 5, Table S3). Interestingly, a large proportion (98%) of the total DEGs were significantly up/downregulated in the WT strain (|Log_2_FC| ≥ 2) while silent in ∆*BbSlt2* (|Log_2_FC| ≤ 0.5) in response to acute heat stress (Figure 5), indicating *B. bassiana* failed to regulate suites of key genes with loss of MAP kinase Slt2 to buffer cells from acute heat stress. Of the DEGs upregulated in in the WT strain (while silent in ∆*BbSlt2* in response to acute heat stress), fungal membrane functioning and basal metabolism were significantly enriched, encoding membrane proteins (173), hydrolases (123), transporters (63), peptidases (34), and carbohydrate metabolism-associated proteins (35) (GO, *p* < 0.001) (Figure 7A). All 24 downregulated DEGs refer to genes upregulated in the WT strain while silent in ∆*BbSlt2* under acute heat stress and significantly enriched in catalogs of mitochondrial proteins (4), transferases (4), and FAA biosynthesis (3) (Figure 7A). KEGG annotation revealed that the responses of a suite of pathways to acute heat stress were disrupted in ∆*BbSlt2.* Of these, six genes in fatty acid biosynthesis were mobilized in the WT strain bearing acute heat stress, with increased expression of four acyl transferases and downregulation of three other key enzymes, but those genes failed to respond in ∆*BbSlt2* under heat stress treatment (Figure 7C). Cell-wall integrity-related pathways (glycosaminoglycan degradation (5) and glycan degradation (5)) were also significantly enriched (*p* < 0.01) in DEGs downregulated in the WT strain while silent in ∆*BbSlt2* under acute heat stress treatment.

According to the curated algorithm for DEG discovery described the Section 2, ∆*BbSlt2* yielded a total of 473 DEGs, with 269 relatively upregulated and 204 downregulated as compared to the WT parent amended with sustained heat treatment (12 h at 32 °C) (Figure 5, Table S3). Similar to acute stress treatment, almost 40% of all DEGs (188) were differentially expressed in the WT strain while silent in ∆*BbSlt2* under sustained heat stress, indicating a paralyzed response of ∆*BbSlt2* to heat stress. Functional annotation of all DEGs showed that vast stress adaptation-associated pathways or gene families were significantly enriched, including hydrolases (51), peptidases (21), and the ribosome biogenesis pathway (16) in upregulated DEGs and oxidoreductases (39), carboxylic acid metabolism [14], glutathione metabolism (5), and sulfur metabolism (6) in downregulated DEGs (GO, *p* < 0.01) (Figure 7B). ROS detoxification was attenuated in ∆*BbSlt2*, with decreased expression of three GSH-S-transferases (BBA_03339, BBA_07410, and BBA_08746) and three Cyp450 enzymes (BBA_04832, BBA_03828, and BBA_09588) as compared to the WT strain under sustained heat stress. Meanwhile, the lipid metabolism was also paralyzed by two enoyl reductases (BBA_05177 and BBA_00966), four lipases/esterases (BBA_08812, BBA_08164, BBA_05788, and BBA_06086), three acyl-CoA dehydrogenases (BBA_02637, BBA_00097, and BBA_05669), and an Enoyl-CoA hydratase (BBA_08000), which were downregulated in ∆*BbSlt2* bearing sustained heat stress (Figure 7D, Table S3). The cell-wall integrity process was also disrupted, with three genes associated with cell-wall maturation (an endo-1,3-beta-glucosidase (BBA_00046), the Asp allergen (BBA_06285), and one LysM protein (BBA_08602)), Mad1 adhesin, and two core genes (BBA_09587/Dit1 and BBA_09588/CYP56C1) responsible for siderophore (dityrosine) biosynthesis failing to be mobilized in ∆*BbSlt2*. Basal amino acid catabolism was also affected, with five key genes relatively upregulated and four genes downregulated in ∆*BbSlt2* under sustained heat stress (Figure 7E). Of the 107 overlapped DEGs under both acute and sustained heat stress, 2 genes were co-downregulated, and 99 DEGs were symmetrically upregulated, which included suites of cell wall-associated proteins: glycoside hydrolase (7) (annotated by CAZY) and transporters (32) (annotated by TCDB).

### 3.6. Deletion of BbMpk1 Adversely Enhanced Fungal Resistance to Oxidative Stress via Regulation of Suites of Key Pathways

**Figure 8:**
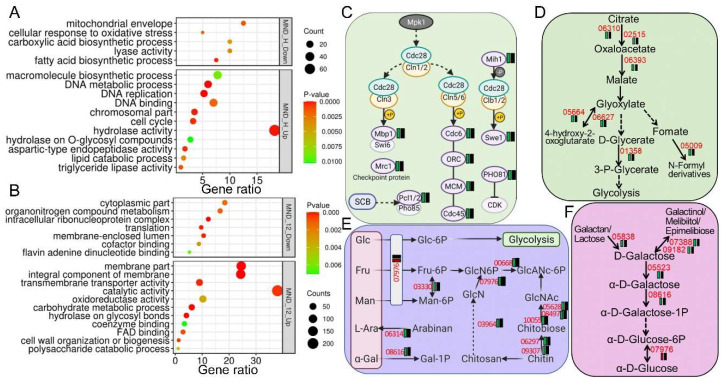
GO enrichment analysis and illustration of over-represented metabolic pathways of DEGs collected from WT and ∆*BbMpk1* strains treated with menadione stress as compared to non-stress conditions. Go term enrichment of DEGs in ∆*BbMpk1* as compared to the WT parent under 12 h (**A**) or 30 min (**B**) of osmotic stress when normalized to non-stress conditions. (**C**) Expression profile of DEGs in the cell-cycle process under 30 min of MND-generated oxidative stress (gene tagged as yeast homolog). (**D**–**F**) Comparative expression of DEGs in glyoxylate and dicarboxylate metabolism (**D**), Amino sugar and nucleotide sugar metabolism (**E**), Galactose metabolism (**F**) under 12 h oxidative stress treatment when compared to non-stress conditions. Annotations of expression and gene IDs are the same as in Figure 6.

Ablation of BbMpk1 also desensitized the fungal response to acute oxidative stress (0.5 h menadione). Only 26 out of 441 DEGs (5.9%) were greatly upregulated in ∆*BbMpk1*, while others were silent in ∆*BbMpk1* but significantly upregulated (374) or downregulated (40) in the WT strain (Figure 5, Table S4). The WT strain responsively downregulated suites of fundamental metabolism, including DNA metabolism (24), sets of hydrolases (73), and lipid catabolism (6), but failed to respond in ∆*BbMpk1* (Figure 8A). Similarly, the WT strain mobilized genes in the mitochondrial envelope, including two cytochrome c/c1 heme lyases (BBA_02809/BBA_09495), the COX17 protein (BBA_00190), HAD phosphatase (BBA_02721), and peptidase M16C (BBA_07823); those involved in monocarboxylic acid biosynthesis, i.e., two fatty acid synthases (FASs) (BBA_06886 and BBA_06887) and the Elo1 fatty acid elongation protein (BBA_08363) in response to acute oxidative stress, were silent in ∆*BbMpk1* (Figure 8A). The impact of BbMpk1 on fungal mitochondrial (MT) function was tested with JC-1 fluorescence staining of MT membrane potential (Figure S1B). Our data show that under acute oxidative stress, MT aggregates (high membrane potential) in ∆*BbMpk1* were more likely to be scattered inside the cell and yield more monomers (low membrane potential) as compared to the WT parent, indicating a significant loss of MT membrane potential after deletion of BbMpk1. Interestingly, the cell-cycle pathways downstream of the Mpk1 kinase were largely downregulated (10 genes) in the WT strain but unchanged in ∆*BbMpk1* in response to acute oxidative stress, indicating an unchanged running of the cell cycle (Figure 8C). Four differentially expressed genes—two upregulated (BBA_07277 and BBA_03790) and two downregulated (BBA_06693 and BBA_06336)—were randomly selected from the ∆*BbMpk1* mutant for qRT-PCR validation under acute oxidative stress. Their expression profiles were consistent with the transcriptomic analysis (Figure S1D).

**Figure 9:**
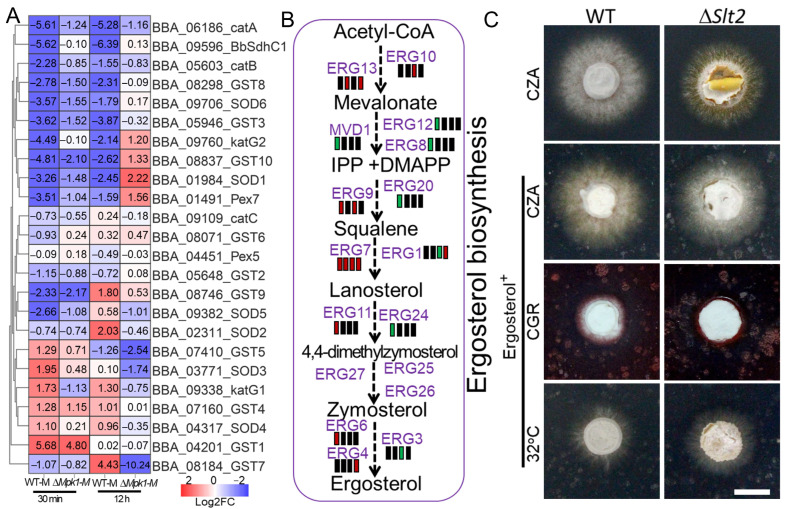
Expression profiles of genes in ROS detoxification-, ergosterol biosynthesis, and ergosterol-supplemented phenotypical tests of ∆*BbSlt2*. (**A**) Relative expression of catalases, SODs, and GSTs in ∆*BbMpk1* and the WT strain under MND stress. (**B**) Differential expression patterns of genes in the ergosterol biosynthesis pathway under heat stress; four rectangles beside gene names refer to the expression pattern under 30 min heat stress (WT, ∆*BbSlt2*) and 12 h heat stress (WT, l ∆*BbSlt2*). Red, black, and green indicate upregulation, no change, and downregulation, respectively, as compared to non-stress conditions. (**C**) Phenotypical growth of the WT and ∆*BbSlt2* strains on the indicated medium supplemented with 2.5 mM ergosterol (bar indicates 10 mm).

Collectively, the sustained oxidative stress treatment identified 669 DEGs that were relatively upregulated (555) or downregulated (114) in ∆*BbMpk1* as compared to the WT parent (Figure 5, Table S4). A few of the key oxidative stress-associated pathways were greatly upregulated in ∆*BbMpk1*, including membrane proteins (136), suites of oxidoreductases (57), glycosyl-hydrolases (23), and cell wall biogenesis (7) (GO, *p* < 0.01) (Figure 8B). Ablation of BbMpk1 also relatively downregulated sets of nitrogen metabolic pathways of organonitrogen metabolism (19), RNA translation (11), and the ribonucleoprotein complex (14). Interestingly, similar to acute oxidative stress, ∆*BbMpk1* showed unchanged activity of fundamental metabolism, with increased expression of suites of enzymes in glyoxylate and dicarboxylate metabolism (Figure 8D), amino/nucleotide sugar metabolism (Figure 8E), galactose metabolism (Figure 8F), and ROS detoxification, e.g., MSF transporters (26), oxidoreductases (57), FAD binding proteins (16), and cytochrome P450 (4) as compared to the sensitive wild-type strain (Table S4). Otherwise, expression of genes involved in pathways impacting organonitrogen compounds and those involved in GABA biosynthesis were also elevated in the WT strain but not significantly changed in ∆*BbMpk1* when examining the 12 h menadione DEG datasets. Of the 97 co-regulated DEGs in ∆*BbMpk1* bearing 30 min/12 h menadione stress, 95 DEGs were co-upregulated (88) or co-downregulated (7) as compared to the unstressed WT strain. Six MFS transporter and five protease/peptidase and member genes in oxidative phosphorylation (BBA_09373 and BBA_09596) were co-upregulated in ∆*BbMpk1* (Table S4). In contrast, two genes in the mitochondria integrity and respiration chain (BBA_00190/COX17 protein and BBA_02809/cytochrome c/c1 heme lyase) and two transferase genes (BBA_08686 and BBA_08685) were co-downregulated in ∆*BbMpk1* when compared to the WT parent under acute/sustained oxidative stress. As a key member of the respiratory chain, succinate dehydrogenase unit C (BbSdhC1) contributes to ROS homeostasis and oxidative stress in *B. bassiana* [24]. Our data showed that BbSdhC1 was significantly downregulated in the WT strain under 30 min (Log_2_FC = −5.6) or 12 h (−6.4) of MND stress but unchanged in ∆*BbMpk1* (30 min/−0.1, 12 h/0.13) as compared to non-stress treatment. The expression of ROS detoxification-associated genes, including catalases (CAT/katG), superoxide dismutase (SOD), and glutathione S-transferases (GST) [11,25], were screened. The expression levels of three catalases, three GST genes, and two SOD genes were downregulated in the WT strain while silent or upregulated in ∆*BbMpk1* when exposed to MND stress (30 min/12 h). Furthermore, one katG gene, five GST genes, and four SOD genes were also differentially expressed in ∆*BbMpk1* as compared to the WT strain under MND stress (30 min/12 h) (Figure 9A). Several membrane transporters and ion exchangers also played key roles in fungal relief of oxidative stress in *B. bassiana*; peroxisomal import proteins Pex5 and Pex7 [26] were downregulated in the WT strain (−3.5) and ∆*BbMpk1*(−1.0) under 30 min of MND stress treatment while depressed in the WT strain (−1.6) but upregulated in ∆*BbMpk1* (1.6) when amended with 12 h MND stress as compared to non-stress conditions.

### 3.7. Crosstalk of MAP Kinases in Regulation of Cell-Wall Integrity and Stress-Associated Processes in Responde to Sensitive Stress Conditions

To identify crosstalk between MAP kinases, the jointly up/downregulated DEGs in all three MAP-kinase mutants bearing acute/sustained stress were screened. Collectively, 45 DEGs were found to be relatively synergistically downregulated (1) or upregulated (44) in all three MAP-kinase mutants as compared to the WT parent, and over 93% of DEGs were greatly up/downregulated in the WT strain and silent in MAPK mutants in response to acute stress treatment (Figure 5, Tables S2–S4). The cell-wall integrity of MAP-kinase mutants was commonly influenced, failing to downregulate 18 secreted protein genes containing 7 peptidases; and 7 membrane-associated proteins, including 4 ion or carbohydrate transporters and 2 glycosidases. The sustained stress treatment yielded 23 DEGs that were synergistically upregulated (19) or downregulated (4) in all MAPK mutants, with over 74% of DEGs differentially expressed in the WT strain while silent in mutants under sustained stress treatments. Of those, two key genes (BBA_02344 and BBA_02345) involved in the biosynthesis of key stress antagonist GABA were upregulated in the WT strain while downregulated in all three MAPK mutants. Suites of cell-wall biosynthesis-associated proteins were relatively upregulated in MAPK mutants, including two types of glycosyl hydrolases (BBA_05838, BBA_08242, BBA_10055, and BBA_05825), three peptidases (BBA_02711, BBA_05879, and BBA_07559) and two lactamase B enzymes (BBA_06483 and BBA_03852) in GSH biosynthesis.

### 3.8. Ergostero- Mediated Membrane Repair Partially Rescues ∆BbSlt2 Sensitivity to Abiotic Stress

The comparative transcriptomic analysis showed that ∆*BbSlt2* yielded 24.1% DEGs under acute heat stress and 18.6% DEGs under sustained heat stress, all of which were cell membrane-associated proteins (Figure 7, Table S3), indicating a significant impact on membrane integrity of the loss of BbSlt2 that aggravated the fungal heat sensitivity. Visualization of ergosterol biosynthesis also showed the disturbed biosynthesis of ergosterol in ∆*BbSlt2*, with the relative upregulation of upstream genes of ERG13, ERG12, ERG8, MVD1, ERG20, and ERG24 and downregulated of suites of downstream genes of ERG9, ERG11, and ERG6 under acute heat stress. Sustained heat stress abnormally elevated the expression of ERG13, ERG1, ERG3, and ERG4 but depressed the expression of ERG10 in ∆*BbSlt2* as compared to the WT parent (Figure 9B). As key a lipid in the fungal membrane, ergosterol is known to rescue defects in cell-membrane integrity and abiotic stress resistance in *B. bassiana* [17]. A phenotypical growth test of ∆*BbSlt2* with supplementation of ergosterol (0.25 mM) was carried out (Figure 9C). Interestingly, the collapse of colony growth of ∆*BbSlt2* was rescued by the addition of ergosterol with similar colony morphology to that of the WT parent. The aggravated growth defects of ∆*BbSlt2* under cell wall-perturbing stress (Congo red) and heat stress (32 °C) were also erased with the addition of ergosterol (Figure 1A and Figure 9C). All these results indicate that the addition of ergosterol partially rescued ∆*BbSlt2* membrane damage and phenotypical growth with/without abiotic stress.

## 4. Discussion

MAPK cascades convergently evolved as part of the fungus kingdom and are essential for fungal development, stress response, and host–microbe interactions, and the roles of three MAP kinases in stress responses were unveiled in insect pathogenic fungi, with specialized roles in pathogenesis and stress resistance [5,12,14,15,27]. However, the regulation networks of MAP kinases responding to specialized stress conditions and the crosstalk between MAP kinases had not been fully investigated in entomopathogenic fungi. Our study revealed the regulatory network mediated by Hog1, Slt2, and Mpk1 MAP kinases as highly specialized stress response commanders in *Beauveria bassiana*: Hog1 is indispensable for osmotic adaptation, Slt2 for thermal and cell-wall integrity, and Mpk1 for oxidative stress resilience. Time-course RNA-seq and WGCNA identified 12 co-expression modules (547 genes), highlighting core processes—cell-cycle, glycolysis, cytoskeletal dynamics, and MAPK signaling pathways—regulated by a singular MAP kinase under specialized stress (acute/sustained) treatment. Comparative transcriptomic analysis uncovered the fact the main metabolic and signal pathways mobilized by MAP kinases echo specialized stress environments. These insights deepen our theoretical understanding of fungal stress–response networks and pinpoint MAP kinase-regulated nodes for the engineering of superior biocontrol strains and the dissection of pathogen–host interactions.

Consistent with previous reports in model fungi and entomopathogens, the Hog1 cascade in *B. bassiana* was found to be indispensable for osmotic stress tolerance [14,27]. Our analysis showed that the deletion of Hog1 in *B. bassiana* greatly altered cell morphology in cell-surface and cell-wall carbohydrates and attenuated fungal growth under osmotic stress, indicating an essential role of the Hog1 cascade in fungal development and osmotic stress tolerance. The Δ*Bbhog1* mutant exhibits markedly reduced levels of erythritol and arabinitol under osmotic stress, which are crucial for maintaining cellular osmotic balance (14). Our data also indicated that the ablation of BbHog1 downregulated three key genes in glycolysis, and the glycolysis stress assay showed decreased glycolysis activity of Δ*Bbhog1* in response to acute osmotic stress. Therefore, the defected glycolysis metabolism in Δ*Bbhog1* may negatively disrupt the biosynthesis of erythritol and arabinitol in the mutant. ConA and WGA antigens labeled carbohydrates on the cell wall and the expression of cell wall-associated processes, including cell-wall integrity, polysaccharide metabolism, and chitosan biosynthesis, were also decreased in ∆*BbHog1* as compared to the WT strain under osmotic stress, indicating a potential domino effect of MAP kinase Hog1 on the Slt2 cascade in regulating cell-wall integrity. Suites of lipid metabolic pathways, including glycerophospholipid metabolism, lipid metabolism, and ketone-body metabolism, were also disrupted with the loss of Hog1, indicating that an alternative lipid-mediated cell turgor generation pathway may exist to facilitate fungal resistance to osmotic stress [16]. The core MAP-kinase signal pathway was also disrupted, with two downstream regulators (Tup1/Ctt1) of Hog1 downregulated and the expression of three other components of the MAP-kinase pathway abnormally elevated in Δ*Bbhog1* in response to acute osmotic stress. Comparative transcriptomic analysis revealed that the metabolic processes of suites of Hog1 regulating new metabolic pathways, including amino acids (tryptophan and valine/leucine/isoleucine), downregulated, while members of cell skeleton dynamics and suites of CYP450s were abnormally upregulated in ∆*BbHog1* under acute/sustained osmotic stress treatment. These results indicate that the Hog1 cascade mobilized diverse pathways and generated complex regulatory networks, enabling the fungus to maintain homeostasis under osmotic stress conditions.

The Slt2 cascade was found to be crucial for the regulation of cell-wall integrity (CWI) maintenance and repair in response to cell wall-perturbing stress agents (Congo red and calcofluor white) and thermal stress in both yeast and filamentous fungi [8,28,29]. Deletion of Slt2 in *B. bassiana* yielded a weakened cell wall, decreased content or activity of cell wall-associated glycoproteins and enzymes, and deteriorated fungal growth under cell wall-perturbing stress [15,18,30]. The abnormal filiform textured hyphae of ∆*BbSlt2* and decreased ConA and WGA antigens labeled types of carbohydrates under heat stress also indicated an essential role of BbSlt2 in fungal cell-wall integrity. ∆*BbSlt2* showed severe colony collapse compared to the WT strain and other two MAPK mutants on SDA plates at 32 °C. The deletion of Slt2 also paralyzed fungal response to acute heat stress while greatly changing the global expression profile under sustained heat stress as compared to the WT parent, highlighting a master role of Slt2 in heat resistance other than cell-wall stress. Under acute thermal stress, ∆*BbSlt2* yielded 718 DEGs, with a striking 98% of those being heat-responsive in the WT strain but transcriptionally silent in ∆*BbSlt2*. Those dysregulated pathways were enriched in membrane proteins, hydrolases, transporters, lipid metabolism, oxidoreductases, and cell-wall remodeling, indicating that Slt2 not only regulates the CWI pathway but also governs multifaceted stress-responsive networks that are essential for fungal survival. Ergosterol is essential for cellular membrane integrity and plays vital role in environmental stress response, especially the response to thermal stress [31,32,33,34]. Ergosterol metabolism was found to be severely impaired in ∆*BbSlt2*, leading to possible membrane instability, and ergosterol supplementation partially rescued ∆*BbSlt2* growth under heat and cell-wall stress, indicating that Slt2 positively regulates membrane sterol balance, a phenomenon also observed in lipid-deficient strains of *B. bassiana* [17]. These results indicate a robust role of Slt2 in regulating metabolic adaptability and redox defense under thermal pressure, offering a new molecular explanation for fungal heat-stress response and ecological adaptation to host insects in entomopathogenic fungi.

The Fus3 (Mpk1) MAP-kinase cascade is known to regulate fungal sexual/asexual development and filamentous growth in yeast and filamentous fungi [13]. Pathogenic fungi adopt Mpk1 (Fus3) for the regulation of host penetration and appressorium formation [5,12,35]. In addition to its roles in development and pathogenesis, Fus3 (Mpk1) has emerged as a key mediator of fungal stress responses, orchestrating gene expression and signaling networks that enable adaptation to diverse environmental challenges. The results of our phenotypic test coincide with those of previous studies that found that the deletion of BbMpk1 paralyzed host cuticle penetration and increased tolerance to oxidative stress in *B. bassiana* [5,23]. The abnormal protrusions on the cell surface and increased WGA antigen-tagged N-acetylglucosamine signal on the cell surface of ∆*BbMpk1* indicated an increase in cell-wall ingredients after loss of BbMpk1, which may somehow contribute to oxidative stress resistance by shielding cells from exogenous oxidative radical damage via an enhanced cell wall [7,36]. Transcription factors BbOsrR2 and BbOsrR3 were found to mediate fungal oxidative stress response by regulating the Fus3-MAP kinase pathway in *B. bassiana* [37]. Transcriptomic analysis revealed that the BbMpk1 MAP kinase plays a pivotal role in mediating *Beauveria bassiana*’s response to oxidative stress by orchestrating transcriptional reprogramming of multiple protective and metabolic pathways. Upon acute oxidative challenge (0.5 h MND), the ∆*BbMpk1* mutant displayed a severely blunted transcriptional response, with only 5.9% of differentially expressed genes (DEGs) upregulated, while the wild-type (WT) strain exhibited robust regulation of 414 DEGs, including significant repression of DNA metabolism, hydrolase activity, and lipid catabolism, alongside the induction of mitochondrial envelope- and monocarboxylic acid biosynthesis-related genes. The increased accumulation of MT membranal monomers in ∆*BbMpk1* also indicated decreased membrane potential with the loss of BbMpk1. These results suggest that BbMpk1 is essential for activating mitochondrial functions and metabolic suppression under stress, likely to limit ROS generation and preserve cellular integrity [11,25]. Furthermore, the failure of ∆*BbMpk1* to downregulate cell-cycle genes indicates an inability to arrest proliferation under stress, potentially exacerbating ROS damage, consistent with the conserved role of Mpk1 in cell-cycle regulation under stress in fungi [8]. Under prolonged oxidative stress (12 h), the ∆*BbMpk1* mutant activated alternative detoxification pathways, such as the overexpression of MFS transporters, oxidoreductases, and cytochrome P450s, reflecting a compensatory but possibly less efficient ROS mitigation mechanism. Notably, BbSdhC1, a key mitochondrial component linked to ROS homeostasis [24], was sharply downregulated in the WT strain but unaltered in ∆*BbMpk1*, implicating BbMpk1 in suppressing mitochondrial respiration to reduce ROS load. Similarly, canonical antioxidant genes, including catalases, GSTs, and SODs, were transcriptionally repressed in the WT strain under stress, a response that was either alleviated or reversed in ∆*BbMpk1*, indicating disrupted fine-tuning of ROS scavenging systems. These findings highlight the fact that BbMpk1 confers oxidative stress resistance by integrating transcriptional networks involving respiration, metabolism, and detoxification, reinforcing its potential as a genetic target for improved fungal fitness and biocontrol efficacy in hostile environments.

## 5. Conclusions

This study establishes a comprehensive mechanistic framework of the Hog1, Slt2, and Mpk1 MAPK cascades as specialized and interconnected stress-response hubs in *Beauveria bassiana*. By integrating phenotype assays, time-resolved transcriptomics, WGCNA, and comparative pathway analysis, we revealed that each MAPK acts as a dedicated commander for osmotic, thermal/cell-wall, and oxidative types of stress adaptation while jointly shaping metabolic homeostasis, redox balance, and cell-wall remodeling. Our findings uncover previously unrecognized regulatory circuits—including glycolysis-dependent osmoadaptation, Slt2-mediated sterol homeostasis, and Mpk1-controlled cell-wall remolding and mitochondrial reprogramming—and demonstrate extensive MAPK crosstalk that fine-tunes fungal fitness under acute and sustained stress treatments. This work not only expands the conceptual understanding of MAPK specialization and coordination in entomopathogenic fungi but also provides actionable molecular targets for the engineering of robust biocontrol strains and the deciphering of host–pathogen interactions in complex environmental niches.

## Data Availability

The raw RNA-seq data were deposited in the NCBI Gene Expression Ominibus with series accession number PRJNA865046.

## Supplementary Materials

The following supporting information can be downloaded at: https://www.mdpi.com/article/10.3390/jof11120839/s1, Figure S1: Three types of fluorescent visualization and qRT-PCR validation of differentially expressed genes in MAP-kinase mutants under the indicated stress conditions; Table.S1: Oligonucleotides used in this study; Table.S2: Annotation of differentially expressed genes in ∆BbHog1 versus the WT strain under time-course (0, 0.5, or 12 h) osmotic stress (1.2 M sorbitol) treatment. Sob-0.5 and Sob-12 indicate groups of DEGs under osmotic stress for 0.5/12 h versus non-stress treatment, respectively; Table.S3: Annotation of differentially expressed genes in ∆BbSlt2 versus the WT strain under time-course (0, 0.5, or 12 h) heat stress (32 °C) treatment. HT-0.5 and HT-12 indicate groups of DEGs for 0.5/12 h heat stress treatment, respectively, versus treatment at 26 °C; Table.S4: Annotation of differentially expressed genes in ∆BbMpk1 versus the WT strain under time-course (0, 0.5, or 12 h) oxidative stress (16 μM menadione) treatment. MND-0.5 and MND-12 indicate groups of DEGs for 0.5/12 oxidative stress treatment, respectively, versus non-stress conditions.
