# Supplementary material for: MAPK Pathways Coordinate Stress Adaptation by Mobilizing Specialized Gene Modules in Entomopathogenic Fungus Beauveria bassiana
Source: J Fungi (Basel). 2025 Nov 27;11(12):839. doi: 10.3390/jof11120839 (PMC12734328; doi:10.3390/jof11120839)
Supplement: Supplementary file 1 [file jof-11-00839-s001.zip › Figure S1.pdf]

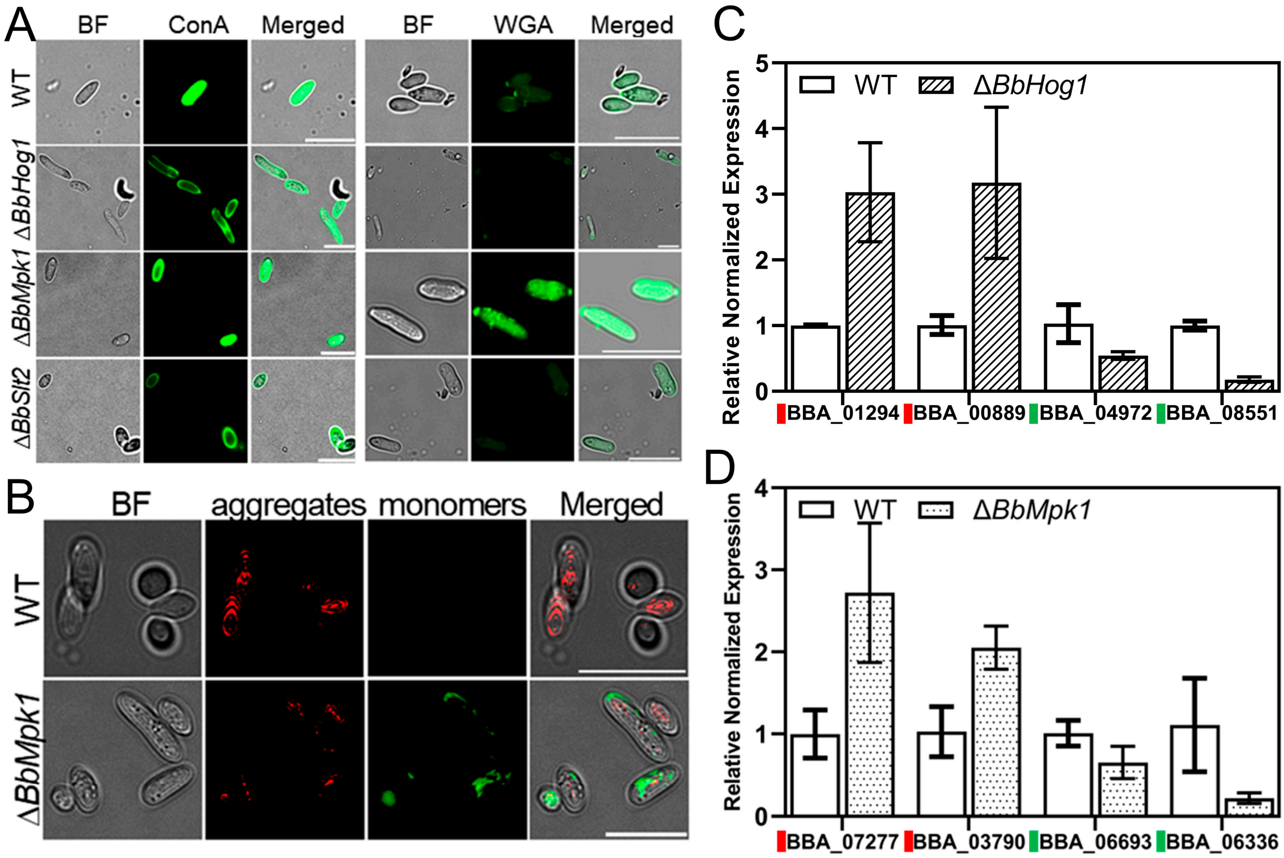

Figure. S1 Three types of fluorescent visualization and qRT-PCR validation of differentially expressed genes in MAP-kinase mutants under the indicated stress conditions. A, Observation of the blastospores of three MAP-kinase mutants and WT strains transiently treated with given stress treatments (30 min) and marked with ConA and WGA antigens. Bar indicates 10 μm. B, JC-1 staining of mitochondrial membrane potential of WT and Δ*BbMpk1* blastospores after treatment with menadione for 30 min. Aggregates and monomers are visible as red and green fluorescence, respectively. Bar indicates 10 μm. C and D, qRT-PCR validation of randomly selected differentially expressed genes in Δ*BbHog1* and Δ*BbMpk1* treated with transient osmotic or oxidative stress treatments, respectively. Red and green boxes beside gene names indicate up/downregulation DEGs, respectively, in mutants as compared to the WT parent.
